# Individual and generalized lower limb muscle activity and kinematic adaptations during walking on an unpredictable irregular surface

**DOI:** 10.1186/1757-1146-7-S1-A3

**Published:** 2014-04-08

**Authors:** Charlotte Apps, Rui Ding, Jason Tak-Man Cheung, Thorsten Sterzing

**Affiliations:** 1 Sports Science Research Center, Li Ning (China) Sports Goods Co Ltd, Beijing 101111, China; 2 School of Sport and Exercise Sciences, Liverpool John Moores University, Liverpool, L3 3AF, UK

## Background

Natural surfaces are irregular but limited studies have researched their effect on gait because of the predominantly flat surfaces where measurements are taken [1]. Regularly, biomechanical research also tends to group mean results of many subjects together to find the generalised response to a constraint. This often hides individual adaptation strategies [2]. Therefore, the purpose of this study was to analyse biomechanical responses during walking on an unpredictable irregular surface (UIS), at the individual level.

## Methods

Seventeen healthy, male participants (mean (SD): 24.2 (1.6) years, 1.76 (0.05) m, 71.6 (8.2) kg) walked on a treadmill at 5 km/h with a predictable regular surface (PRS) and on UIS, created by attaching EVA dome shaped inserts of different heights (10 mm and 15 mm, both in diameter of 140 mm) and hardness (40 and 70 Asker C). The mean and standard deviation, as a measure of variability, were calculated for lower limb kinematics and electromyography of five selected muscles of the left leg for 16 steps. Single parameters between individuals were compared, and additionally, group results between the treadmill surfaces were obtained by Wilcoxon signed ranked tests (p<.05).

## Results

**Figure 1:**
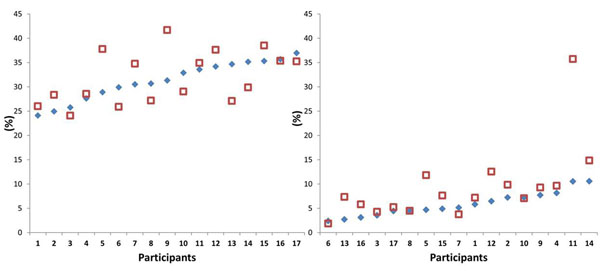
Normalized mean EMA of gastrocnemius medialis: Individual response during stance phase (left) indicated by no systematic relationship between surfaces, generalized group response during latency period (right) indicated by increased EMA on UIS for vast majority of participants. Squares denote irregular surface, diamonds regular surface.

The step mean and step variability of EMG muscle activation (EMA) of thigh muscles, vastus medialis and bicep femoris, were specific to the individual, with no systematic alterations between surfaces. In contrast, shank muscles showed individual and common EMA strategies depending on the period of the gait cycle (Figure 1). During total stance phase, mean EMA of the tibialis anterior and gastrocnemius medialis altered individually. However, within the muscular latency period (first 30 ms after foot strike) mean EMA strategies were no longer individual. There was a common group decrease and increase observed in mean EMA of the tibialis anterior and gastrocnemius respectively on UIS. Conversely, kinematic characteristics appeared rather common between participants throughout. Mean and especially variability of sagittal and frontal plane ankle angles, as well as knee and hip characteristics were similar on PRS and UIS between individuals.

## Conclusion

Individual thigh muscle EMG responses were accompanied by similar kinematics between participants when walking on UIS. Thus, mainly the surface constraint altered movement kinematics, although underlying muscle activation strategies were individualised. For a better understanding of adaptations to shoe-surface interactions, next to searching for common neuro-muscular patterns during the whole gait cycle or total stance phase, more focus should be placed on individual analyses of sub-periods of stance phase.
